# Promoting childbirth companions in South Africa: a randomised pilot study

**DOI:** 10.1186/1741-7015-5-7

**Published:** 2007-04-30

**Authors:** Heather Brown, G Justus Hofmeyr, V Cheryl Nikodem, Helen Smith, Paul Garner

**Affiliations:** 1 Worthing Hospital, Lyndhurst Road, Worthing, W Sussex, BN2 DH, UK; 2 Effective Care Research Unit, University of the Witwatersrand/University of Fort Hare/East London Hospital Complex, South Africa; 3 Faculty of Community and Health Sciences, University of the Western Cape, South Africa; 4 International Health Group, Liverpool School of Tropical Medicine, Pembroke Place, Liverpool L3 5QA, UK

## Abstract

**Background:**

Most women delivering in South African State Maternity Hospitals do not have a childbirth companion; in addition, the quality of care could be better, and at times women are treated inhumanely. We piloted a multi-faceted intervention to encourage uptake of childbirth companions in state hospitals, and hypothesised that lay carers would improve the behaviour of health professionals.

**Methods:**

We conducted a pilot randomised controlled trial of an intervention to promote childbirth companions in hospital deliveries. We promoted evidence-based information for maternity staff at 10 hospitals through access to the World Health Organization Reproductive Health Library (RHL), computer hardware and training to all ten hospitals. We surveyed 200 women at each site, measuring companionship, and indicators of good obstetric practice and humanity of care. Five hospitals were then randomly allocated to receive an educational intervention to promote childbirth companions, and we surveyed all hospitals again at eight months through a repeat survey of postnatal women. Changes in median values between intervention and control hospitals were examined.

**Results:**

At baseline, the majority of hospitals did not allow a companion, or access to food or fluids. A third of women were given an episiotomy. Some women were shouted at (17.7%, N = 2085), and a few reported being slapped or struck (4.3%, N = 2080). Despite an initial positive response from staff to the childbirth companion intervention, we detected no difference between intervention and control hospitals in relation to whether a companion was allowed by nursing staff, good obstetric practice or humanity of care.

**Conclusion:**

The quality and humanity of care in these state hospitals needs to improve. Introducing childbirth companions was more difficult than we anticipated, particularly in under-resourced health care systems with frequent staff changes. We were unable to determine whether the presence of a lay carer impacted on the humanity of care provided by health professionals.

**Trial registration**: Current Controlled Trials ISRCTN33728802

## Background

In South Africa, the quality of care for women during childbirth could be improved: women are often left alone for long periods during childbirth, and in some instances women are shouted at, struck, or slapped [1]. Staff are under stress due to lack of resources, and this demoralises the work force [2]. However, women will not use a service they judge to be of poor quality [3], and choosing not to deliver in a health facility can compromise maternal and foetal outcomes [4].

Continuous support for women during labour has several benefits: women are less likely to need intrapartum analgesia, operative birth, or to report dissatisfaction with their childbirth experiences [5]. The Cochrane review reported that continuous support during labour was associated with greater benefits in trials where the companion was not a member of the hospital staff, when the availability of the companion began early in labour, and in settings where epidural analgesia was not routinely available [5]. Despite the availability of this evidence, health professionals have been slow to implement companion policies and programs to ensure that women in labour have appropriate companionship. This is particularly the case in low- and middle-income countries such as South Africa, where companions during childbirth are not commonly encouraged by state maternity services.

We hypothesised that the presence of a childbirth companion could, in addition to these beneficial effects on labour, also influence provider behaviour, functioning as an independent witness and community observer. We considered this might thus improve the quality of care, and promote a more women-friendly service. We conducted a pilot cluster randomised trial that aimed to promote childbirth companions using an educational intervention targeted at health staff on site. The main objective was to pilot and evaluate a multi-dimensional educational intervention, directed at maternity staff promoting childbirth companions. A subsidiary objective was to detect if the intervention had a large effect on practice: the study was not designed to detect small or moderate changes in practice.

## Methods

### Study hospitals

**Figure 1:**
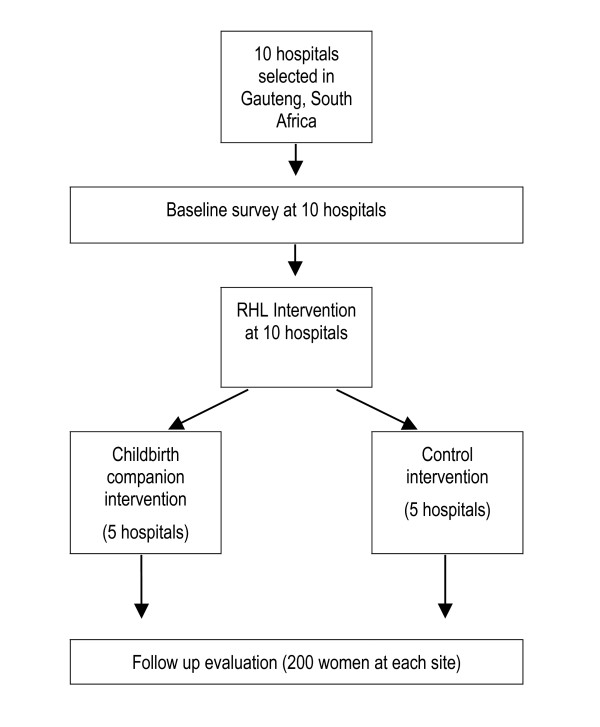
Study flow diagram.

We selected 10 maternity services that consistently had more than 80 deliveries per month from a list of services providing maternity care within a 200 km radius of Johannesburg, Gauteng Province, South Africa. These services included midwife obstetric units (MOUs), district hospitals with medical generalists and caesarean section facilities available (level 1 hospitals) and referral hospitals with obstetric specialists and caesarean section facilities available (level 2 hospitals). Hospitals linked to university academic departments were excluded. Figure 1 illustrates the overall study design.

### Randomisation

The hospitals were matched in pairs according to number of deliveries per month and whether facilities for caesarean section were available. One of each pair was randomly selected to receive the childbirth companion intervention. Randomisation within pairs was conducted out of the country (Liverpool, UK) by a researcher who was blind to the facility characteristics (Table 1).

**Table 1:** Characteristics of study hospitals

		Pair 1	Pair 2	Pair 3	Pair 4	Pair 5
Intervention	Level of care	Level 1	MOU	MOU	Level 2	Level 2
	Deliveries per month	180	150	200	800	500
Control	Level of care	Level 1	Level 1/MOU	MOU	Level 2	Level 2
	Deliveries per month	130	130	65	870	400

MOU: midwife obstetric unit; Level 1: medical generalists and caesarean section available; Level 2: referral hospital with obstetric specialists and caesarean section available.

### Intervention

Prior to the childbirth companions intervention, all 10 hospitals were provided with access to the WHO Reproductive Health Library (RHL) [6] and were given a training session on how to use it. This was carried out over two months in 1999. Five hospitals were then allocated to the childbirth companion intervention; a multidimensional educational package, implemented at the hospitals in the subsequent two months. This intervention was directed at maternity staff and aimed to increase the number of women who had a companion during childbirth. Materials were developed by the researchers to promote childbirth companions, and consisted of: (a) an interactive workbook for use in a special workshop for doctors and midwives; (b) colour posters and banners for the labour ward encouraging women to bring a companion with them; (c) illustrated pamphlets informing staff and pregnant women how the maternity unit could promote childbirth companions; (d) a magazine-style video programme on companionship that used interviews with women who had recently given birth in South African maternity services, and maternity staff (available from the WHO Reproductive Health Library). The workshop and materials provided detailed suggestions on how to promote childbirth companions. Initially, this was by providing evidence on the benefits of childbirth companions; it also covered how to decide what would be the most appropriate choice of childbirth companion in a particular setting (partners, volunteers, family members), how to recruit volunteer childbirth companions (including training the companions to 'praise, comfort and support women' during childbirth), and how to identify and address obstacles to promoting childbirth companions. The video programme (in both CD ROM and VHS formats) was left at the hospitals. A programme to track the use of RHL was installed on each machine, but due to technical problems this did not record log-ins reliably and the data could not be used in the analysis.

Hospital superintendents and lead consultants for obstetrics and gynaecology at each hospital were visited prior to baseline data collection; project objectives were outlined and permission to conduct the study was obtained. All hospitals agreed to participate and support the study. The same staff members were re-visited prior to implementing the intervention and were encouraged to attend the workshop sessions; at all sites the superintendent, head midwife or lead consultant attended the first workshop. The first author (HB) made two special visits to each site: the first visit was to introduce the concept of childbirth companions to the maternity staff and the second visit to hold the 3 hour workshop, together with other members of the team (JH, VCN, HS), making use of the special materials that had been developed by the researchers. HB subsequently visited each facility every two weeks to encourage implementation by discussing progress with staff and ways to overcome obstacles to change.

The five control hospitals received an unrelated evidence-based intervention to promote external cephalic version (ECV) [7]. This was to ensure that all of the hospitals included in the project received an intervention and a similar number of visits from the research team so that we could evaluate the effect of the intervention rather than the effect of participating in a research project. This 'control' intervention comprised a lecture by a locally-known expert on evidence related to ECV, group discussion, a video demonstration, and an invitation to attend training in ECV with HB; two staff members made appointments, but neither actually attended.

### Process evaluation

As we were testing the materials and the process of implementing the intervention, the main investigator also kept a diary, completed after every visit, documenting the endeavours of the staff to implement childbirth companionship.

### Outcomes

The primary outcome measure was whether a woman was allowed a companion with her during childbirth. Secondary outcomes were (a) other beneficial policies (being allowed to move around in the 1st and in the 2nd stage of labour); (b) painful obstetric policies where benefit doubtful (routine episiotomy, routine enema); (c) indicators of humane care (being offered food, being offered water); and (d) indicators of inhumane care (being shouted at, being slapped or struck, being left alone).

### Data collection

At each site, we conducted 200 baseline exit interviews with postnatal women (excluding those delivered by elective caesarean section) from October 1998. We used the same interview schedule at follow up 8 months after the intervention. Women were interviewed by one of a team of field workers using a structured interview schedule, either in English or in their home language if they did not speak English (the field workers all spoke two or more of South Africa's eleven official languages). The field workers were trained by one of the researchers (VCN) and quality control checks on their interviews conducted, followed by weekly meetings to resolve problems. Quality control was performed halfway through each of the interviewing periods by one of the researchers (VCN or HB).

### Sample size and data analysis

The cluster sample size of ten hospitals was determined according to the capacity of the research team hospitals available in the study area, and took into account that this was a pilot study to explore use of the materials. This was deemed sufficient to pilot the intervention, detect major effects, and explore the dynamics of implementing the intervention, whilst understanding that the study was relatively underpowered to detect modest or moderate effects. For data analysis, data clerks entered the data from interviews with postnatal women into Epi Info 6 (version 6.04, Atlanta, GA: Centers for Disease Control and Prevention; 2001.). Range checks on all parameters were performed. The units of comparison were the hospitals, and change from baseline to follow-up at each site was calculated. Median changes were compared between the study and the control hospitals, using the one tailed Mann-Whitney U test.

### Ethical approval

The Committee for Research on Human Subjects (Medical) of the University of the Witwatersrand (Protocol Number M960512); Gauteng Provincial Health Department and the administrator at each site approved the study.

## Results

### Feasibility of implementation

From the diary of the investigator, we are able to describe the process of implementation. Of the five hospitals randomised to the childbirth companion intervention, staff at four decided to implement the program. At follow-up visits to these four hospitals, the childbirth companion posters, banners and pamphlets were evident and childbirth companions could be seen with some women.

At one of the four hospitals (a midwife obstetric unit) volunteer childbirth companions were recruited from the community, trained and started to provide companionship to women during labour. The training consisted of a single session with the volunteers where their role in praising, comforting and supporting the women during childbirth was clarified using discussion and role play. All the volunteers apart from one had had children themselves. The decision to use volunteer supporters at this site was made by staff as they thought there was not sufficient space or privacy in the labour ward to accommodate childbirth companions of the women's choice. At this site four to five women delivered in one room with no partitions or screens. The volunteer childbirth companions were initially popular, but they reported that it was difficult to find money for transport to the unit. There was an attempt by the maternity staff to raise funds but this did not continue. After the Christmas break the volunteers did not come back. They sent messages to say that it was too expensive for them. Hence all forms of childbirth companionship ceased.

The second site, also a midwife obstetric unit, had previously allowed childbirth companions under special circumstances, for example, when teenagers delivered. They were able to increase the use of childbirth companions after the intervention, as each woman is allocated an individual cubicle. They did not use volunteer childbirth companions as they perceived that the woman attending their service were all able to bring a companion of their choice if told in advance.

The third and fourth hospitals (both level one hospitals) allowed childbirth companions, but restricted them when the labour ward was very busy and women were sharing cubicles for delivery. One of these hospitals additionally recruited a single volunteer companion during the study period.

At the fifth companion intervention site (a level two hospital) the maternity staff initially agreed to introduce childbirth companions. However, due to internal problems at the hospital (the maternity unit was due to be moved to another nearby health service) they were unable to initiate this. However, they did initiate a childbirth companion program in the regional midwife obstetric units, which referred to the hospital maternity unit. Childbirth companions were not allowed to accompany the woman if she was transferred during labour to the hospital. The programme at the regional midwife obstetric units is not reflected in the postnatal interviews, which were limited to women giving birth at the hospital.

### Evaluation

Table 1 shows the characteristics of the hospitals and Table 2 the women interviewed in the pre- and post-intervention surveys. A total of 2090 women were interviewed at baseline. Table 3 shows baseline practices across all ten study hospitals. Most women (84%) had not had a childbirth companion; many reported that food (77%) and fluids (84%) were withheld during childbirth. Almost half of the women (47%) were not allowed to move about during the first stage of labour, and most (97%) were restricted to a supine position during the second stage of labour. Episiotomy was performed on 35% of women, and 53% received an enema. Women reported being shouted at (18%), and slapped or struck (4%). When asked about what they thought about the care they had received, 5% described the care as bad or very bad.

**Table 2:** Characteristics of women sampled in the pre and post intervention surveys

Outcomes	Childbirth companionship package included (intervention hospitals)						Childbirth companion package not included (control hospitals)				
Women interviewed	Before	215	199	204	214	207	203	204	207	206	231
	After	200	221	211	215	209	204	200	199	199	200
Age (years) Mean (SD)	Before	25.8(5.74)	26.0 (5.48)	25.9 (5.54)	26.7 (6.78)	25.9 (6.3)	26.4 (6.85)	26.2 (5.8)	25.4 (5.07)	25.5 (6.37)	28.2 (6.43)
	After	26.2 (5.9)	25.8 (5.82)	26.5 (5.62)	26.6 (6.15)	26.1 (6.4)	25.9 (6.6)	26.4 (5.89)	26.0 (5.62)	25.5 (6.14)	27.1 (6.81)
Gestation (wks) Mean (SD)	Before	39.5 (1.7)	39.9 (0.91)	39.9 (0.53)	38.0 (3.22)	39.4 (2.29)	38.4 (2.25)	39.4 (1.94)	40 (0.197)	39.5 (1.95)	39.1 (2.65)
	After	39.3(1.24)	37.8 (1.57)	39.8 (0.81)	36.8 (2.9)	38.9 (1.73)	37.3 (2.3)	39.4 (2.05)	40 (0.24)	39.4 (1.76)	39.9 (2.63)

**Table 3:** Reported practices across all ten hospitals at baseline

**Practice**	**%**	**N**
Not allowed companion	84.5	2085
Preferred partner as companion	49.7	2056
Left alone	16.2	2080
Not allowed food	77.4	2089
Not allowed fluids	83.6	2089
Moving around not allowed during first stage of labour	46.6	2083
Moving around not allowed during second stage of labour	97.1	2072
Episiotomy performed	34.7	2073
Enema given	52.9	2087
Shouted at	17.7	2085
Slapped or struck	4.3	2080
Care described by woman as bad or very bad	5.3	2089

### Impact evaluation

2058 exit interviews were carried out 14 months after the baseline survey and eight months after the RHL and childbirth companions intervention had been introduced. No effect was demonstrated on the number of women having a companion; and no effect was demonstrated on being shouted at, left alone, not offered food or fluids or physically mistreated (Table 4). There was a statistically significant reduction in episiotomy and fewer women reporting being mobile during the second stage of labour at the intervention hospitals compared with control hospitals, although this was one of many variables tested for significance.

**Table 4:** Women's report of change in practice before and after the intervention in intervention and control hospitals

**Category**	**Outcomes**	**Group**	**Before intervention**	**After intervention**	Change: paired comparison*
Primary outcome	Companion allowed	EXPT	9 (5 to 45)	21 (0 to 40)	
		CONTROL	2 (1 to 60)	15 (0 to 48)	NS

Other beneficial policies	Moving around in 1st stage	EXPT	56 (51 to 66)	64 (55 to 86)	
		CONTROL	51 (40 to 61)	54 (51 to 64)	NS
	
	Moving around allowed in 2nd stage	EXPT	2 (1 to 9)	1 (0 to 4)	
		CONTROL	2 (0 to 3)	32 (1 to 39)	P = 0.003

Painful obstetric policies	Episiotomy	EXPT	34 (25 to 48)	21 (10 to 28)	
		CONTROL	40 (21 to 44)	39 (24 to 48)	P = 0.02

Benefit doubtful	Enema given	EXPT	56 (33 to 63)	52 (35 to 68)	
		CONTROL	66 (18 to 69)	41 (19 to 72)	

Humane care	Offered food	EXPT	25 (2 to 37)	19 (0 to 46)	
		CONTROL	26 (4 to 34)	23 (13 to 40)	NS
	
	Offered fluid	EXPT	18 (3 to 29)	14 (11 to 28)	
		CONTROL	10 (0 to 31)	16 (13 to 25)	NS

Inhumane care	Shouted at	EXPT	14 (6 to 25)	15 (10 to 22)	
		CONTROL	21 (12 to 25)	26 (12 to 29)	NS
	
	Slapped or struck	EXPT	2 (2 to 8)	1 (0.5 to 4)	
		CONTROL	3 (2 to 12)	4 (0.5 to 7)	
	
	Left alone	EXPT	12 (7 to 21)	16 (4 to 32)	
		CONTROL	14 (11 to 29)	21 (10 to 33)	NS

* Mann-Whitney U test.

NS: No statistically significant difference.

## Discussion

The baseline data derived from the interviews with over 2000 post natal women describes a level of care for women during childbirth in ten South African state maternity services that is far from ideal. This is similar to results from previous research into care during childbirth in South Africa [1] but the large numbers of women interviewed in this study mean that our results have a depth of information that has not been described previously. Overall, across the hospitals the care of women during childbirth is not in line with current best practice. Most women are not allowed companions during labour [5] and are not encouraged to move around during the first stage [8] or second stage of labour [9], despite clear evidence of the benefit of these practices. There is a high rate of episiotomy despite good evidence that routine episiotomies are not of benefit and may do harm [10]. In addition, unpleasant practices such as not being allowed food or drink and being given an enema are common.

Women reported being shouted at or slapped or struck. Despite these examples of particularly poor care, only 5% of women felt that the care they received was bad or very bad. This suggests that women have a low expectation of quality of care when they attend maternity services.

In introducing childbirth companions, we had not appreciated the level of organisation required, and the need for a substantive shift in organisation by the staff working on labour wards. There was a willingness to proceed with this policy and initial evidence of implementation, but by the time the follow-up investigation was conducted there was no evidence of change in practice. It seems that despite a willingness to implement childbirth companions, policies, practical restrictions and adverse health service conditions, such as transfer of staff implementing the programmes out of the maternity unit, militated against sustained success. Implementation was apparent at several hospitals: for example, one centre made considerable progress in recruiting community volunteer childbirth companions, however the system broke down after the Christmas break, again influenced by staff turnover. Unfortunately we did not interview staff, which may have provided more insight into the obstacles in promoting childbirth companions.

Observing the number of women who actually had a companion with them during childbirth might have been a more accurate measure of the success of the intervention. However, because we were concerned with influencing policy in this area, we thought it was important to ask women if they were allowed a companion; this allows for the possibility of women declining the offer of a support person based on personal preference. Another limitation is that we again did not have the resources to carry out qualitative interviews with women or observation to determine if there were other obstacles. For example, women may have detected subtle or non-verbal reasons why they perceived that they were not allowed a childbirth companion. We would recommend others conducting similar research measure whether a companion was allowed, as well as a companion actually being present, to document more reliably any change in maternity staff practice.

Better quality care – both by making it evidence-based and more humane – is likely to improve utilisation of maternity services. Studies in Zimbabwe showed that that non-use of services is associated with poor foetal and maternal outcomes [3] and that the pattern of utilisation of maternity services by rural women was based on rational decision making that took into account not only the distance to a service but also whether the care provided was seen to be good [11]. South African women describe preferring to stay at home to deliver their babies rather than attend services [1]. Hence there is a need to address the quality of obstetric care in government facilities to improve the uptake of services and outcomes in poorer groups. We had hoped that by implementing childbirth companions, the number of instances of poor care of women in terms of shouting, slapping and striking would be reduced. This was perhaps unrealistic and simplistic. The reasons why, for example, labour ward staff abuse patients have been explored at length by Jewkes et al [2] and is a highly complex issue.

We found that an educational programme, while apparently well accepted by the staff, did not appear to have substantive, sustained effects on practice. This is consistent with previous research on educational strategies to change practice [12]. The secondary changes observed, a reduction in the number of women having episiotomies and reporting being mobile in the second stage of labour, could be the result of multiple significance testing, or confounders such as other training programmes running simultaneously.

Changing clinical practice is recognised to be difficult to achieve and likely to require multifaceted approaches, such as recruiting support from opinion leaders, academic detailing, involving health providers in objective setting, and audit and feedback [13]. Interventions to influence practitioner practice have not been tested extensively in developing countries, and little is known about what works in resource poor settings and why [14]. Because of the difficulties in replicating and documenting complex interventions, they are often evaluated using phased or non-experimental designs that provide information about context and critical success factors, as well as the likelihood that change will be sustained beyond the study period [15,16].

For these reasons we have expanded the educational strategies used in this pilot study to develop the 'Better Births' Initiative (BBI), promoting evidence-based, humane care during labour [17,18]. In addition to instruction on the principles of evidence-based care and providing evidence regarding labour practices, the BBI programme uses workshops to allow labour ward staff to identify changes in practice they regard as important, to set goals for change, and to use an audit and feedback mechanism to monitor progress (see Additional files 1 and 2). A pilot of this intervention was conducted in Gauteng province specifically to study qualitatively the critical factors influencing diffusion of knowledge into changed behaviour [19]. This study identified trends towards good practice six months after implementing the educational programme, but found the changes in practice to be quite random. Sometimes the motivation to change came from one individual; at other hospitals, it required a team consensus. Practice change was consistently more likely for procedures that could be stopped easily (routine enemas and perineal shaving) and less likely for procedures that required additional resources or time to implement (reducing use of the supine position, encouraging companionship).

## Conclusion

This pilot study clearly demonstrated that implementation programmes for childbirth companions need further development and testing. Researchers can draw on this experience in the development of future interventions and particularly in ensuring that in testing such interventions robust outcomes are used to measure the effect of the intervention. In addition, we suggest that more consideration should be given to seeking political commitment from health authorities and senior staff within hospitals, and public awareness campaigns to promote greater sustainability.

## Competing interests

The author(s) declare that they have no competing interests.

## Authors' contributions

The project was initiated by GJH, VCN and PG. GJH, VCN, PG and HB developed materials, HB (with GJH, VCN and HS) implemented the project. HB, VCN and GJH analysed the data. All authors contributed to the writing of the manuscript.

## Pre-publication history

The pre-publication history for this paper can be accessed here:



## Supplementary Material

Additional file 1 A workbook used in workshops with midwives as part of the Better Births Initiative; this material was developed and published in 2001. Please note the systematic reviews underpinning these clinical procedures outlined in the materials could be out of date. We would recommend that the current evidence for each intervention or policy is reviewed before promoting practice based on these materials. For up to date Cochrane systematic reviews in sexual and reproductive health relevant to developing countries please refer to the current editions of either: the WHO Reproductive Health Library [6] or The Cochrane Database of Systematic Reviews [20]. Click here for file

Additional file 2 A reference booklet for midwives detailing evidence associated with commonly used obstetric procedures; developed as part of the Better Births Initiative and published in 2001. Please note the systematic reviews underpinning the clinical procedures outlined in the materials could be out of date. We would recommend that the current evidence for each intervention or policy is reviewed before promoting practice based on these materials. For up to date Cochrane systematic reviews in sexual and reproductive health relevant to developing countries please refer to the current editions of either: the WHO Reproductive Health Library [6] or The Cochrane Database of Systematic Reviews [20]. Click here for file
