# Supplementary material for: Promoting childbirth companions in South Africa: a randomised pilot study
Source: BMC Med. 2007 Apr 30;5:7. doi: 10.1186/1741-7015-5-7 (PMC1905915; doi:10.1186/1741-7015-5-7)
Supplement: Additional file 1 — A workbook used in workshops with midwives as part of the Better Births Initiative; this material was developed and published in 2001. Please note the systematic reviews underpinning these clinical procedures outlined in the materials could be out of date. We would recommend that the current evidence for each intervention or policy is reviewed before promoting practice based on these materials. For up to date Cochrane systematic reviews in sexual and reproductive health relevant to developing countries please refer to the current editions of either: the WHO Reproductive Health Library [6] or The Cochrane Database of Systematic Reviews [20]. [file 1741-7015-5-7-S1.pdf]

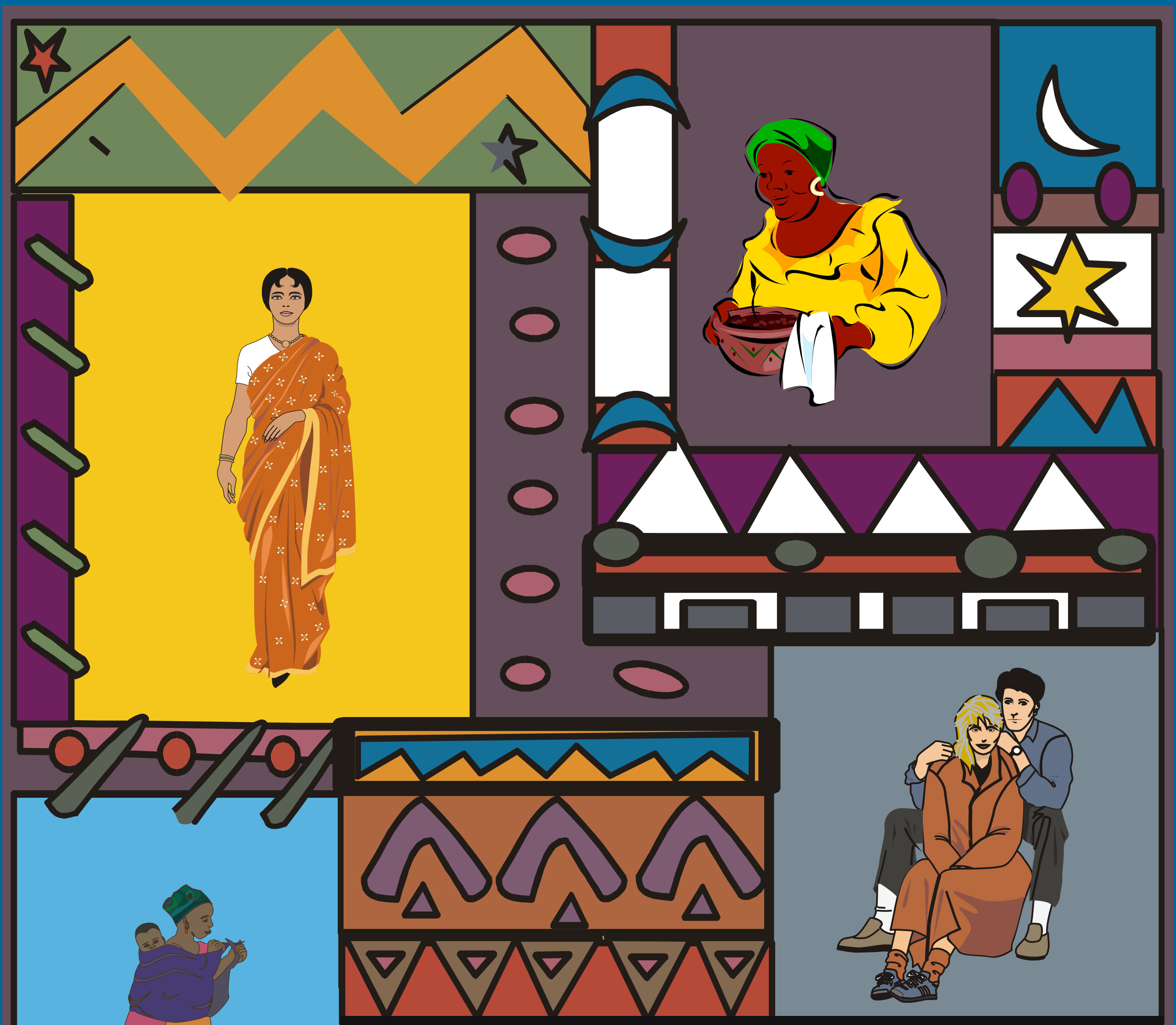

Better Births Initiative

BETTER
BIRTHS
INITIATIVE

MAKING CHILDBIRTH BETTER

A workbook for labour ward staff

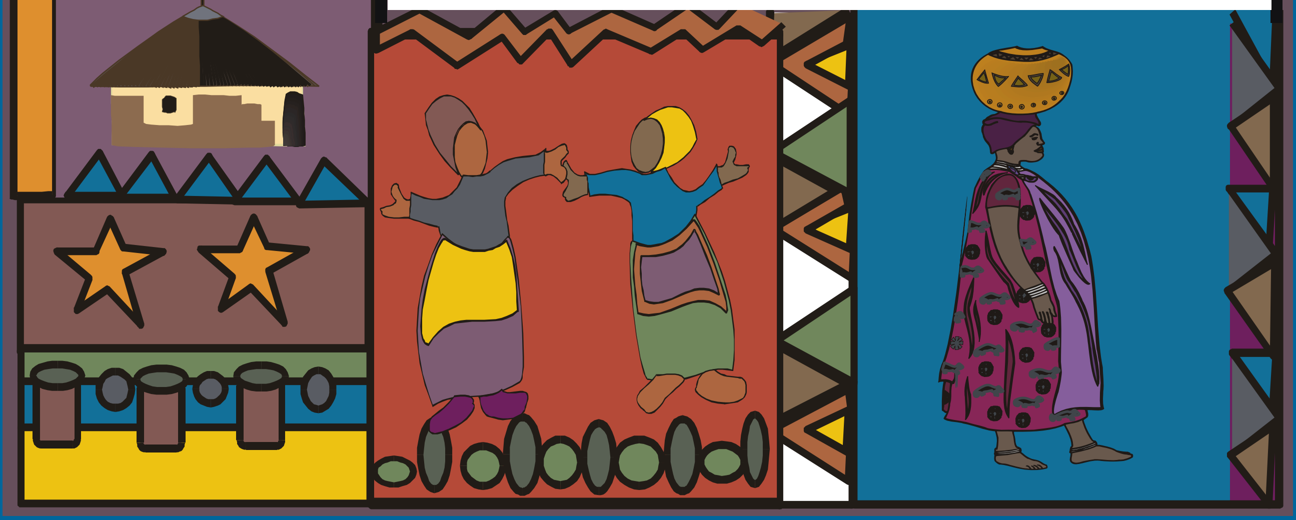

BETTER BIRTHS INITIATIVE

Reproductive Health Research Unit

Effective Care Research Unit

Department of Health, SA

Contact details

Better Births Initiative
Reproductive Health Research Unit
Department of Obstetrics and Gynaecology
Chris Hani Baragwanath Hospital
PO Box Bertsham 2013
Johannesburg

Phone: 011 933 1228
Fax: 011 933 1227
http://www.liv.ac.uk/lstm/bbimainpage.html

Acknowledgements

This book is produced by the Better Births Initiative, a collaboration between: Effective Care Research Unit, University of Witwatersrand, East London Hospital Complex, Liverpool School of Tropical Medicine, UK, Reproductive Health Research Unit, University of Witwatersrand.

Funding: Department for International Development, UK
South African Medical Research Council,
International Childbirth Education Association
Johnson and Johnson

Authors

Helen Smith, Heather Brown, Justus Hofmeyr, Kim Dickson-Tetteh, Paul Garner, Helen Rees

Layout and Design: RHRU Marketing & IEC. Published: March 2001

Contents:

Introduction Page 3

Workshop aims and agenda Page 4

Section 1

Procedures that are used during labour Page 5
Exercise Page 5
Presentation: Best evidence for practices during labour Page 11

Section 2

Moving towards 'Better Births' Page 12
The 'Better Births' standards Page 12
Implementing the 'Better Births' standards in our labour wards Page 13
Exercise Page 14

Section 3
Monitoring practice on the labour ward Page 15
How do we establish a self-audit mechanism in our labour ward? Page 15

Conclusion Page 16
Video presentation: Childbirth
Companions: Every womens choice Page 16

Appendix 1
The best available evidence for procedures used during labour Page 18

Appendix 2
Further information
Pictures of the poster and Self Audit Wall Chart Page 18

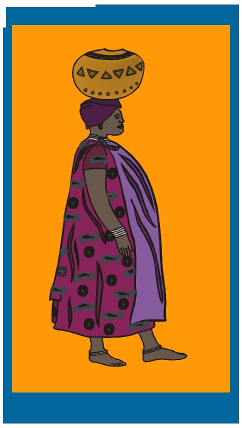

Introduction

More women give birth in our hospitals and clinics than at home these days. In the hospital setting we use many checks and procedures to manage the labour process. These often include things like taking a woman's blood pressure and checking her pulse and temperature. These are done routinely to ensure the health of mother and enable a safe birth. However, there is evidence that some of the procedures used during labour do not actually offer any benefit to women.

Certain procedures are not always necessary but we continue to use them, despite evidence that they can be uncomfortable, wasteful of resources, and even harmful.

Good care should always be based on practices that have been justified by scientific research. As health professionals we must be aware of the most up to date research and be willing to change our practice accordingly. Ideally, only procedures that are necessary for the birth process and are of benefit to women should be used, and those that are potentially harmful or humiliating for women should be stopped. It is often difficult to begin to change practice, especially with our heavy workloads. In this workbook we identify some useful practices to introduce, and also some unnecessary procedures and look at how we can reduce their use. Reducing the use of some procedures can actually save time and resources in a busy labour ward.

A key part of working in labour ward is continuous improvement of the quality of care provided, and making the experience of labour more comfortable for women. Providing good quality and respectful care will not only enhance the reputation of the service, but it will encourage women to attend in good time. This in turn will contribute to improving overall maternal and perinatal health in the country.

The Better Births Initiative (BBI) is a focused set of standards that aim to improve the quality and humanity of obstetric care. The standards are based on the best available evidence, and can be implemented using existing resources. The following principles form the basis of the BBI:

Humanity	-	women treated with respect
Benefit	-	care based on the best available evidence
Commitment	-	health professionals committed to improving care
Action	-	effective strategies to change current practices

This workshop is for labour ward staff who would like to know more about the Better Births Initiative, and how to take the necessary steps towards better care for women during labour. Childbirth is a very important time for a woman and her family. It is essential that we try and make her experience of labour as comfortable and dignified as possible.

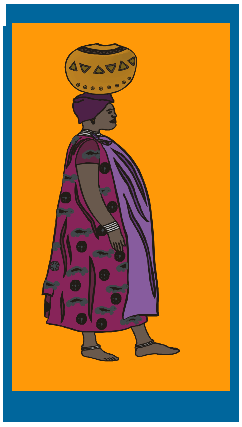

Workshop aims

1. To discuss the benefits and harms of procedures used during labour.
2. To examine research evidence on the benefits and harms of procedures.
3. To discuss ways of changing practice towards 'Better Births'.
4. To provide information on how to implement the four steps to 'Better Births', and set realistic goals for reducing harmful or unnecessary procedures.

Agenda

There are three main sections to the workshop. We aim to complete the workshop in about 2 hours.

We will work through two exercises together, and there will be opportunities for you to contribute to discussions after each one; but comments or questions are welcome at any time during the workshop.

This workbook is for you to keep. Please feel free to write in and to make notes; it is for your reference and it will not be shown to anyone else. The workshop also includes a presentation and video session.

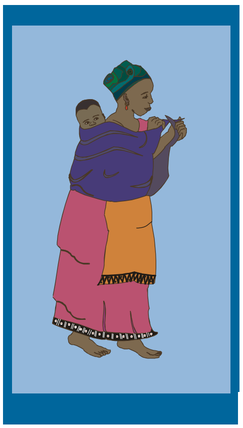

Section 1 - Procedures that are used during labour

In the first section we consider the benefits and harmful effects of different procedures that may be used during labour.

Exercise

The questions below ask you about your own experiences and views in relation to childbirth practice.

- Take up to 10 minutes to read through all the questions and fill in five you find most interesting,
- we will discuss a selection of them together. Additional topics are listed at the end of the exercise, you may wish to discuss them too.
- Please fill in or circle your answers below, and consider only normal vaginal deliveries. The answers are for your use only.

Mobility during labour

1 .At your hospital, during labour are women:

- Expected to stay in bed
- Encouraged to move around freely

2. What benefits and harms do you think are associated with being mobile during labour? Please give an example of each in the table below.

BENEFITS	HARMS

3. Could you comment on the feelings of someone being restricted to bed, either from your own experience, or from imagining yourself in the situation?

Position during birth

1. In what position do most women give birth at your hospital?

- Supine
- Semi-upright (propped up at least 45 degrees with cushions or back rest)
- Lateral (lying on side)
- Upright (squatting or kneeling)

2. What benefits and harms do you think are associated with allowing women to give birth in an upright position? Please give an example of each in the table below.

BENEFITS	HARMS

3.If you were to give birth, which position do you think you would prefer?

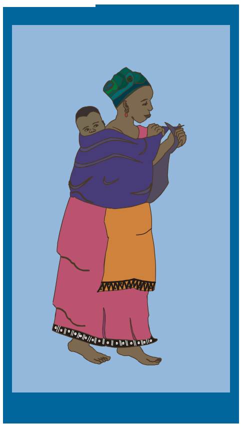

Fluids and food during labour

1. During labour, do you:

- Encourage women to drink fluids as and when they want
- Encourage women to eat if hungry
- Restrict women's intake of fluids
- Restrict women's intake of food

2. What benefits and harms do you think are associated with allowing women to drink fluids during labour? Please give an example of each in the table below.

BENEFITS	HARMS

3. Could you comment on the feelings of someone being kept nil per mouth during labour, either from personal experience, or from imagining yourself in the situation?

Companionship during labour

1. When women gave birth at home, whom did they have with them?

2. In this labour ward, are women often accompanied by:

- Their partner
- A relative or friend
- A community volunteer childbirth companion
- None of the above

3. What benefits and harms do you think are associated with allowing women to have a companion with them during labour? Please give an example of each in the table below.

BENEFITS	HARMS

4. Can you comment from personal experience on the feeling of having someone to hold and comfort you when in pain?

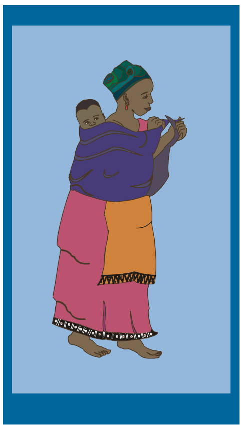

Artificial rupture of membranes

1. At your hospital, is amniotomy, or artificial rupture of membranes used for women in labour?

- Routinely
- In selected cases
- Unsure

2. What benefits and harms do you think are associated with artificially rupturing the membranes routinely?
Please give an example of each in the table below.

BENEFITS	HARMS

Suctioning babies at birth

1. At your hospital, do you suction:

- All babies routinely
- Only babies with meconium
- Unsure

2. What benefits and harms do you think are associated with suctioning all babies?
Please give an example of each in the table below.

BENEFITS	HARMS

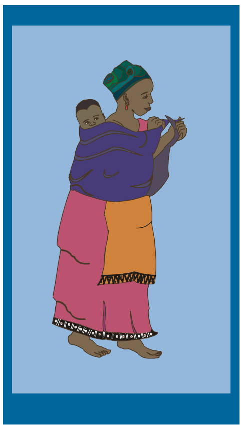

Enema

1. Are enemas given to women during labour at your hospital?

- Unsure
- Never
- Most women
- Certain women

2. What benefits and harms do you think are associated with giving an enema? Please give an example of each in the table below.

BENEFITS	HARMS

3. If you were to give birth, would you prefer an enema:

- Routinely
- Not at all
- Only if constipated
- Other

Shaving

1. At your hospital, are women shaved in preparation for childbirth?

- Unsure
- Never
- Most women
- Certain women

2. What benefits and harms do you think are associated with shaving? Please give an example of each in the table below.

BENEFITS	HARMS

3. Could you comment on the feelings of someone having pubic shaving performed, either from personal experience or from imagining yourself in the situation?

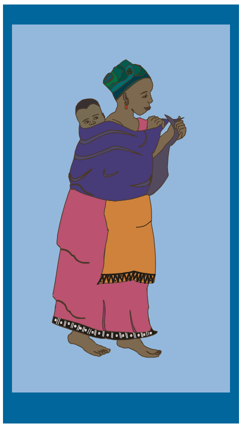

Episiotomy

1. Are episiotomies performed at your hospital?

- Unsure
- Never
- Routinely
- Certain women

2. What benefits and harms do you think are associated with performing episiotomy? Please give an example of each in the table below.

BENEFITS	HARMS

3. Could you comment on the feelings of someone having episiotomy, either from personal experience or from imagining yourself having a similar procedure?

Discussion of benefits, harms and feelings associated with the different procedures.

Other topics you may wish to discuss

There may be some procedures, not mentioned in the exercise, that are of particular importance in your setting. You could also discuss these procedures in terms of benefits and harms

* Separation of mother and baby after birth
* Routine use of intravenous fluids
* Magnesium sulphate for eclampsia
* Oxytocin during third stage of labour
* Strategies to reduce mother to child transmission of HIV

Useful references for each of these procedures appear in Appendix 1 at the end of the workbook.

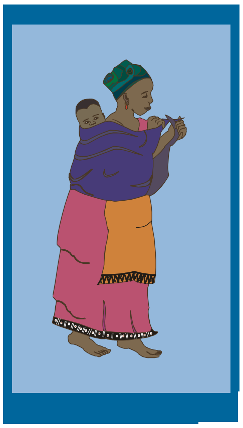

THE BEST EVIDENCE

The first discussion allowed us to discuss what happens in our everyday practice in the labour ward, and what benefits and harms may be associated with using particular procedures. Before making a decision to intervene however, it is important to search for, and be familiar with, the best available evidence regarding benefits and harms associated. Not everyone has time to access this information, the following presentation will summarise relevant research findings.

While listening to the presentation, please fill in points that you feel are important in Chart 1 on the next page.

- There are columns for you to fill in the latest research evidence about benefits and harms.
- Having seen the evidence, do you feel any changes to practice are needed in your hospital?
- Complete the third column by circling 'yes' or 'no' as appropriate
- The chart will be used for discussion, and is for you to keep for your own reference. It will not be shown to anyone else.

CHART 1 - The best evidence

Practice	Benefits	Harmful	Change needed
Mobility during labour			Yes No
Different positions during birth			Yes No
Eating and drinking during labour			Yes No
Companionship during labour			Yes No
Use of enemas			Yes No
Shaving			Yes No
Episiotomy			Yes No
Other practice			Yes No
Other practice			Yes No
Other practice			Yes No

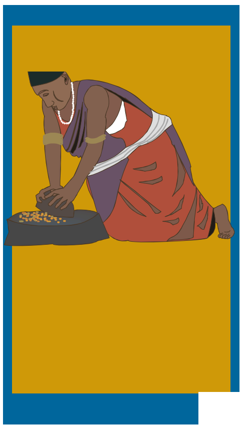

Section 2 - Moving towards 'Better births'

Changing practice is never easy, but it can be done. The following section deals with ways of bringing about change.

To provide good quality care and make the experience of childbirth more comfortable for women we need to ensure that we are guided by the best available research evidence:

- use only those procedures that are appropriate and beneficial

- avoid using procedures that are of no benefit and that may be uncomfortable, humiliating or harmful to women.

Using evidence from clinical trials, research on actual practice, and women's views, the Better Births Initiative has identified changes that can easily be implemented in labour wards in low-income settings

The 'Better Births' standards

This focused set of standards aims to improve the quality and humanity of obstetric care. The standards are based on the best available evidence, and can be implemented using existing resources.

Women can have a 'Better Birth' experience if we:

1. Use procedures that are effective and beneficial:

- Being mobile during labour
- Companionship during labour
- Magnesium sulphate for eclampsia
- Oxytocin in the third stage of labour
- Effective strategies to reduce mother-to-child transmission of HIV

2. Stop using procedures that have no benefit:

- Supine position for birth
- Withholding fluids and food during birth
- Shaving

3. Avoid making interventions routine where there is no evidence of benefit:

- Routine artificial rupture of membranes
- Routine enemas
- Routine episiotomy
- Routine suctioning of neonates without meconium

4. Identify other priority areas in your setting where there is good evidence, and take steps to bring about change. Examples include steroids for preterm delivery, antibiotics for caesarean section, and use of the partogram.

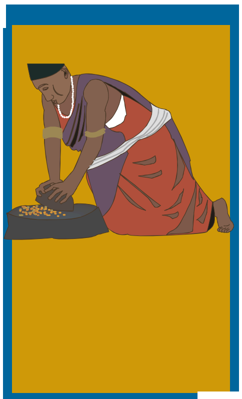

Implementing the 'Better Births' standards in our labour wards

Exercise

Implementing the Better Births standards will require change. Changing practice will involve overcoming barriers, or obstacles, that prevent change from happening. Obstacles may include your own attitudes towards change and beliefs about good practice. The organisation of care and the labour ward environment may also be barriers to change. There will be opportunities, or situations that will encourage change to happen too. These may include updating or revising protocols, Commitment from staff, motivation for change, and communication between all staff.

- Do you think it is possible to promote any (or all) of the Better Births standards in your labour ward?
- Take five minutes to complete chart 2, then we will discuss together obstacles and opportunities for change.
- Decide which standards could be promoted in your labour ward (column one), and fill in the potential obstacles and opportunities for change. An example is provided for the use of upright positions for birth.
- When deciding which standards could be promoted in your setting, you could refer back to chart 1, where you identified changes needed.

Video Presentation: Childbirth Companions: Every Woman's Choice

CHART 2 - Obstacles and opportunities for change

Changes needed	Obstacles to change	Opportunities for change
Example:<br>upright positions for birth	• Staff resistant to change<br>• not trained to conduct Upright delivery	• Communicate with colleagues<br>• Try upright posture on bed for pushing, lie down when head "crowns".

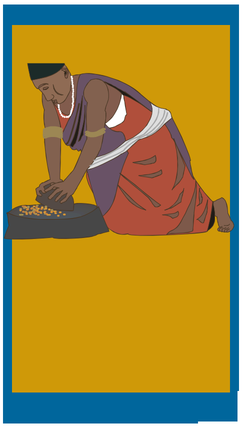

Section 3 - Monitoring practice on the labour ward

The Better Births Initiative is helping labour ward staff to implement evidence-based standards, and make childbirth care more acceptable to women and time and cost efficient. It is difficult to change practice, but it is important to try.

One way that you and your colleagues can introduce change is by using self-audit and feedback. This process can help you examine your current practice for childbirth care. It enables you to conduct a rapid review of the procedures used during labour and delivery, and determine how your practice compares to the ' Better Births standards' (page 14)

To do this, you can use a checklist to record the use of a procedure such as episiotomy for 25 consecutive births. You could display the number of births without episiotomy on a chart and repeat this process regularly, say every 3 months. The results will show you if your practice needs to be changed - for instance if most of the 25 women were given episiotomies, you need to begin to reduce this. After charting the use of episiotomy over several months, you will be able to monitor the progress you have made in reducing the use of episiotomies.

To help in this process, we have designed a checklist and wall charts on which to display the results (see tear out checklist on page 17).

The appraisal is not meant to judge individual performance, therefore staff should be honest when completing the appraisal checklist. Self-appraisal is not self criticism. It is a positive action. Based on the findings of the appraisal, specific action can be taken by staff to promote effective procedures, stop procedures with no evidence of benefit, and avoid using other procedures routinely when they are not always needed.

Displaying the wall charts where all staff can see them will help to motivate you to change.

How do we establish a establish a self-audit mechanism in our labour ward?

1. Make a decision to promote the 'Better Births standards' in your labour ward.

2. Arrange a meeting, or several if you have many staff, to discuss the Better Births Initiative with all the staff. You could use extra workbooks and the presentation and or video, to run your own workshop or discussions.

3. Confirm your commitment to the Better Births Initiative (BBI) by displaying posters, which illustrate the Better births standards, in and around your labour ward.

4. Choose a time to conduct your audit of practice. Elect a team (two or three enthusiastic staff members) who will be responsible for overseeing the recording and charting the use of procedures.

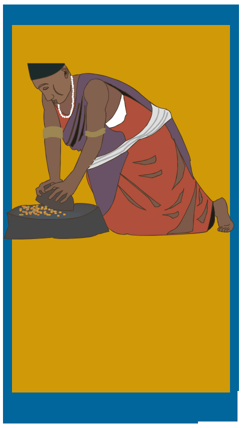

5. Choose which practices you wish to focus on in your hospital. This could include any of the following:

   The number of women who gave birth:
   - being mobile during labour
   - in a position other than supine for birth
   - with adequate food and drink
   - with a companion present
   - without unnecessary suctioning
   - without receiving an enema
   - without pubic shaving
   - without having an episiotomy

   Additionally you may want to monitor the use of intravenous fluids, magnesium sulphate for eclampsia, oxytocin during third stage of labour, strategies to reduce mother to child transmission of HIV, or use of the partogram. Just adapt the wall charts to suit your priorities.

6. Use one wall chart per procedure, and fill in the blank space on the chart with the procedure you want to focus on (outcome chosen box). Pin up the self appraisal wall charts in a central place, where all staff will see them. These will be used to display the use of each procedure and monitor progress.

7. Attach the checklist to the delivery register. For 25 consecutive births, record the use of say, episiotomy, at the time that the maternity register is completed. For simplicity, do not include Caesarean births in the self-audit.

8. After all 25 entries are completed, the appointed team can transfer the results on to the wall charts. Fill in the date of the audit on the chart. As you repeat the audit once a month or 3-monthly, the wall charts will become a permanent feature of the labour ward.

9. Introduce antenatal care staff to the 'Better Births Initiative'. Ask them to display the Better Births' posters in the antenatal clinic.

10. If there is a high turnover of staff in your labour ward, make sure that you find time to introduce new staff members to the Better Births Initiative and the self-audit.

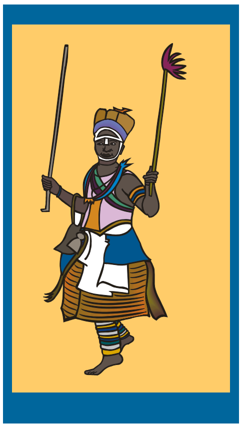

Conclusion

We hope that this workshop and workbook will be of help to each of you. We understand that implementing the 'Better Births' standards and changing the way you practice will not happen quickly.

Moving towards Better Births will take a lot of time, and will depend on your energy and commitment. However, once you have a self-audit mechanism established, it will help you to monitor your progress towards a more evidence-based, humane, and time and cost efficient service.

If you improve the quality of care you provide, then the reputation of your maternity unit will be enhanced and women will be encouraged to attend. The more women appreciate the care they receive, the more satisfaction you will get from your work.

The way forward

Discuss and write down specific actions you will take, the individuals responsible, and the time limits. e.g. meet with hospital administrators; within 2 weeks.

Remember:

- Always consider the potential benefits and harms of the procedures you use and promote the 'Better Births standards' among your labour ward colleagues.
- It is important to involve women in your decisions and to explain to them what you are doing.

Additional materials to help you remember the discussions we have had today:

1. A desktop reference booklet for you to keep. You can refer to this any time to help you remember the benefits of procedures to use.

2. Better Births Initiative poster. If you decide to promote the 'Better Births standards', you can display the posters in the labour ward and antenatal clinic, so that all staff and women can read them.

3. Self-Audit wall charts. One can be put up in the labour ward for each procedure you choose to monitor

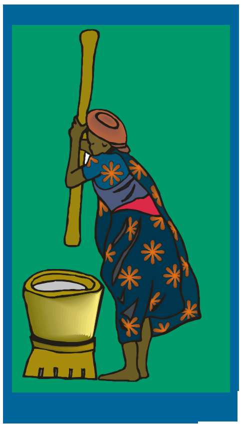

Appendix 1

The best available evidence for procedures used during labour

The evidence of benefit or harm for procedures discussed in this workbook comes from the findings of health care research conducted all over the world. An international organisation - the Cochrane Collaboration - summarises the available research evidence in 'systematic reviews'; these establish what treatments and procedures are of benefit to women, what procedures are harmful, and what is unclear. Below you can find useful references for the procedures mentioned in the workbook. Copies of these papers can be obtained from the workshop facilitator. These are published in the Cochrane library. Many relevant reviews are also published in the WHO Reproductive Health Library (Sec P)

Mobility during labour
1. Department of Reproductive Health and Research, WHO (1999). Care in Normal Birth: apractical guide. Geneva: World Health Organization.
2. World Health Organization. The WHO Reproductive Health Library, issue 4,2001. WHO/RHR/HRP/RHL/3/00. Oxford: Update Software.

Position during birth
1. Gupta JK, Nikodem VC. Woman's position during second stage of labour (Cochrane Review). In: The Cochrane Library, Issue 2, 2001. Oxford: Update Software.
2. Department of Reproductive Health and Research, WHO (1999). Care in Normal Birth: a practical guide. Geneva: World Health Organization.
3. World Health Organization. The WHO Reproductive Health Library, issue 4, 2001. WHO/RHR/HRP/RHL/3/00. Oxford: Update Software.

Fluids and food during labour
1. Department of Reproductive Health and Research, WHO (1999). Care in Normal Birth: a practical guide. Geneva: World Health Organization.
2. Mckay S, Mahan C. Modifying the stomach contents of labouring women: why, how, with what success, and at what risks? How can aspiration of vomitus in obstetrics be prevented? Birth 1988;15 (4):213-221.

Companionship during labour
1. Hodnett ED. Caregiver support for women during childbirth (Cochrane Review). In: The Cochrane Library, Issue 2, 2001. Oxford: Update Software.
2. Department of Reproductive Health and Research, WHO (1999). Care in Normal Birth: a practical guide. Geneva: World Health Organization.
3. Hofmeyr GJ, Nikodem VC, Wolman WL, Chalmers BE, Kramer T. Companionship to modify the clinical birth environment: effects on progress and perceptions of labour, and breastfeeding. Br J Obstetrics and Gynaecology 1991;98:756-764.
4. World Health Organization. The WHO Reproductive Health Library, Issue 4, 2001. WHO/RHR/HRP/RHU3/00. Oxford: Update Software.

Magnesium sulphate for eclampsia
1. Duley L, Henderson-Smart D. Magnesium sulphate versus diazepam for eclampsia (Cochrane Review). In: The Cochrane Library, Issue 2, 2001. Oxford: Update Software.
2. Duley L, Gulmezoglu AM. Magnesium sulphate versus lytic cocktail for eclampsia (Cochrane Review). In: The Cochrane Library, Issue 2, 2001. Oxford: Update Software.
3. Duley L, Henderson-Smart D. Magnesium sulphate versus phenytoin for eclampsia (Cochrane Review). In: The Cochrane Library, Issue 2, 2001. Oxford: Update Software.
5. World Health Organization. The WHO Reproductive Health Library, Issue 4, 2001. WHO/RHR/HRP/RHL/3/00. Oxford: Update Software.

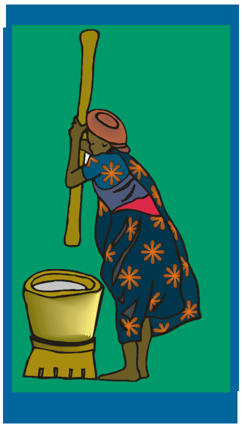

Oxytocin in the third stage of labour
1. World Health Organization. The WHO Reproductive Health Library, Issue 4, 2001. WHO/RHR/HRP/RHL/3/00. Oxford: Update Software.

Strategies to reduce mother to child transmission ofHIV
1. Brocktehurst P. Interventions aimed at decreasing the risk of mother-to-child transmission of HIV infection (Cochrane Review), in: The Cochrane Library, Issue 2, 2001. Oxford: Update Software.
2. Dabis F, Msellati P, Newell ML, Halsey N, Van de Perre P, Peckham C et al. Methodology of intervention trials to reduce mother-to-child transmission ofHIV with special reference to developing countries. AIDS 1995;9 Suppi A:S67-S74.
3. Dunn D, Newell M-L, Ades A, Peckham C. Risk of human immunodeficiency virus type 1 transmission through breast-feeding. Lancet 1992;240:585-8.
4. Landesman SH, Kal'ish LA, Burns DN, M'mkoffH, Fox HE, Zorilla C et al. Obstetrical factors and the transmission of human immunodeficiency virus type 1 from mother to child. N Engi J Med 1996;334:ie17-23.
5. Semba RD, Miotti PG, Chiphangwi JD, Saah AJ, CannerJK, Dallabetta GA et al. Maternal vitamin A deficiency and mother-to-child transmission ofHIV-1. Lancet 1994;343:1593-7.
6. World Health Organization. The WHO Reproductive Health Library, Issue 4, 2001. WHO/RHRMRP/RHL/3/00. Oxford: Update Software.

Artificial rupture of membranes
1. Fraser WD, Turcot L, Krauss I, Brisson-Carrol G. Amniotomy for shortening spontaneous labour (Cochrane Review). In: The Cochrane Library, Issue 2, 2001. Oxford: Update Software.

Enemas during labour
1. Cuervo LG, Rodriguez MN, Delgado MB. Enemas during labor (Cochrane Review). In: The Cochrane Library, Issue 2, 2001. Oxford: Update Software.
2. Department of Reproductive Health and Research, WHO (1999). Care in Normal Birth: a practical guide. Geneva: World Health Organization.
3. World Health Organization. The WHO Reproductive Health Library, issue 4, 2001. WHO/RHR/HRP/RHL/3/00. Oxford: Update Software.

Shaving for delivery
1. Basevi V, Lavender T. Routine perineal shaving on admission in labour (Cochrane Review). In: The Cochrane Library, Issue 2, 2001. Oxford: Update Software.
2. Department of Reproductive Health and Research, WHO (1999). Care in Normal Birth: a practical guide. Geneva: World Health Organization.
3. World Health Organization. The WHO Reproductive Health Library, Issue 4, 2001. WHO/RHR/HRP/RHL/3/00. Oxford: Update Software.

Episiotomy for delivery
1. Carroli G, Belizan J. Episiotomy for vaginal birth (Cochrane Review). In: The Cochrane Library, Issue 2, 2001. Oxford: Update Software.
2. Department of Reproductive Health and Research, WHO (1999). Care in Normal Birth: a practical guide. Geneva: World Health Organization.
3. World Health Organization. The WHO Reproductive Health Library, Issue 4, 2001. WHO/RHR/HRP/RHL/3/00. Oxford: Update Software.

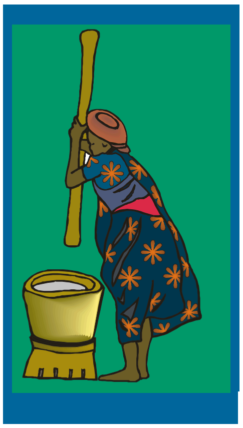

Appendix 2

For further information:

1. Better Births Initiative website:
   Http://www.liv.ac.uk/lstm/bbimainpage.htm.

2. Cochrane Collaboration websites:
   Http://www.update-software.com
   Http://www.cochrane.org/cochrane/ccweb.htm

3. World Health Organization, Reproductive Health Library
   For free subscription contact: Technical Editor
   HRP
   World Health Organization
   1211 Geneva 27
   Switzerland
   Fax: +41 22 791 4171
   Email: RHL@who.ch

4. To search for papers and new evidence, try:
   Http://www.biomedcentral.com/
   Http://www.bmj.com
   Http://www.ncbi.nlm.nih.gov/entrez/querv.fcgi/

Appendix 1
Better Births Initiative - self audit checklist

Checklist to record use of procedures during childbirth.
Use this form to record the use of procedures, for 25 consecutive births, at the time that the maternity register is completed. For simplicity, do not include caesarean births in the self-appraisal. Staff members should fill in this chart immediately after delivery of the baby. Once 25 entries are complete, the totals from the non-shaded columns should be transferred to the wall charts.

You can photocopy this checklist to use on a regular basis (say once every 1-3 months)

Number	Date	Birth register number	Free fluids by mouth		Companion present during labour		Episiotomy		Enema		Suspine position for delivery		Suction of baby (with no meconium)		Other Procedure	
			Yes	No	Yes	No	Yes	No	Yes	No	Yes	No	Yes	No	Yes	No
1																
2																
3																
4																
5																
6																
7																
8																
9																
10																
11																
12																
13																
14																
15																
16																
17																
18																
19																
20																
21																
22																
23																
24																
25																
Totals																

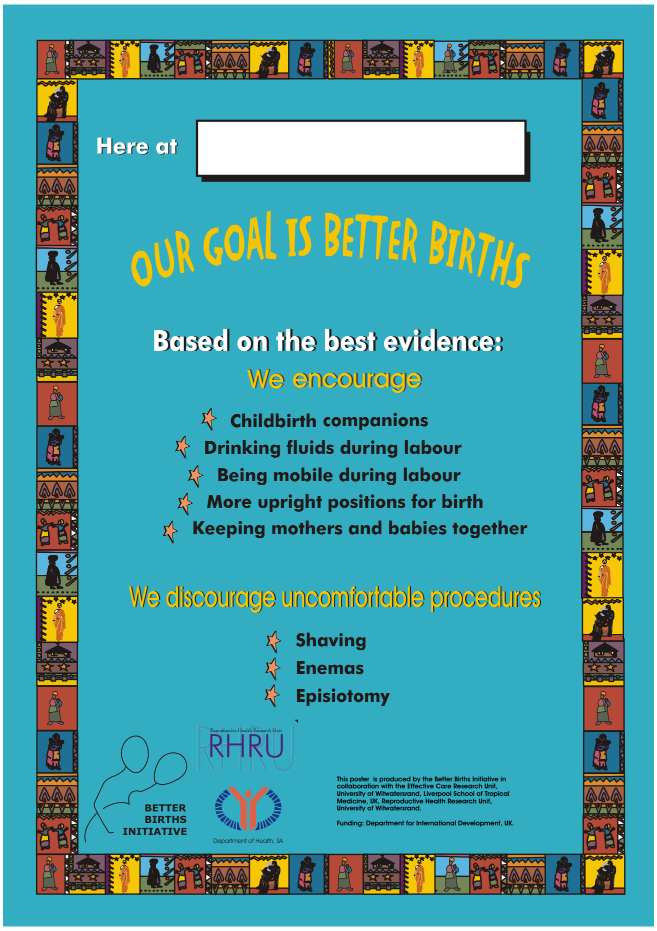
Here at
OUR GOAL IS BETTER BIRTHS
Based on the best evidence:
We encourage
Childbirth companions
Drinking fluids during labour
Being mobile during labour
More upright positions for birth
Keeping mothers and babies together
We discourage uncomfortable procedures
Shaving
Enemas
Episiotomy
Reproductive Health Research Unit
RHRU
BETTER BIRTHS INITIATIVE
Department of Health, SA
This poster is produced by the Better Births Initiative in collaboration with the Effective Care Research Unit, University of Witwatersrand, Liverpool School of Tropical Medicine, UK, Reproductive Health Research Unit, University of Witwatersrand.
Funding: Department for International Development, UK.

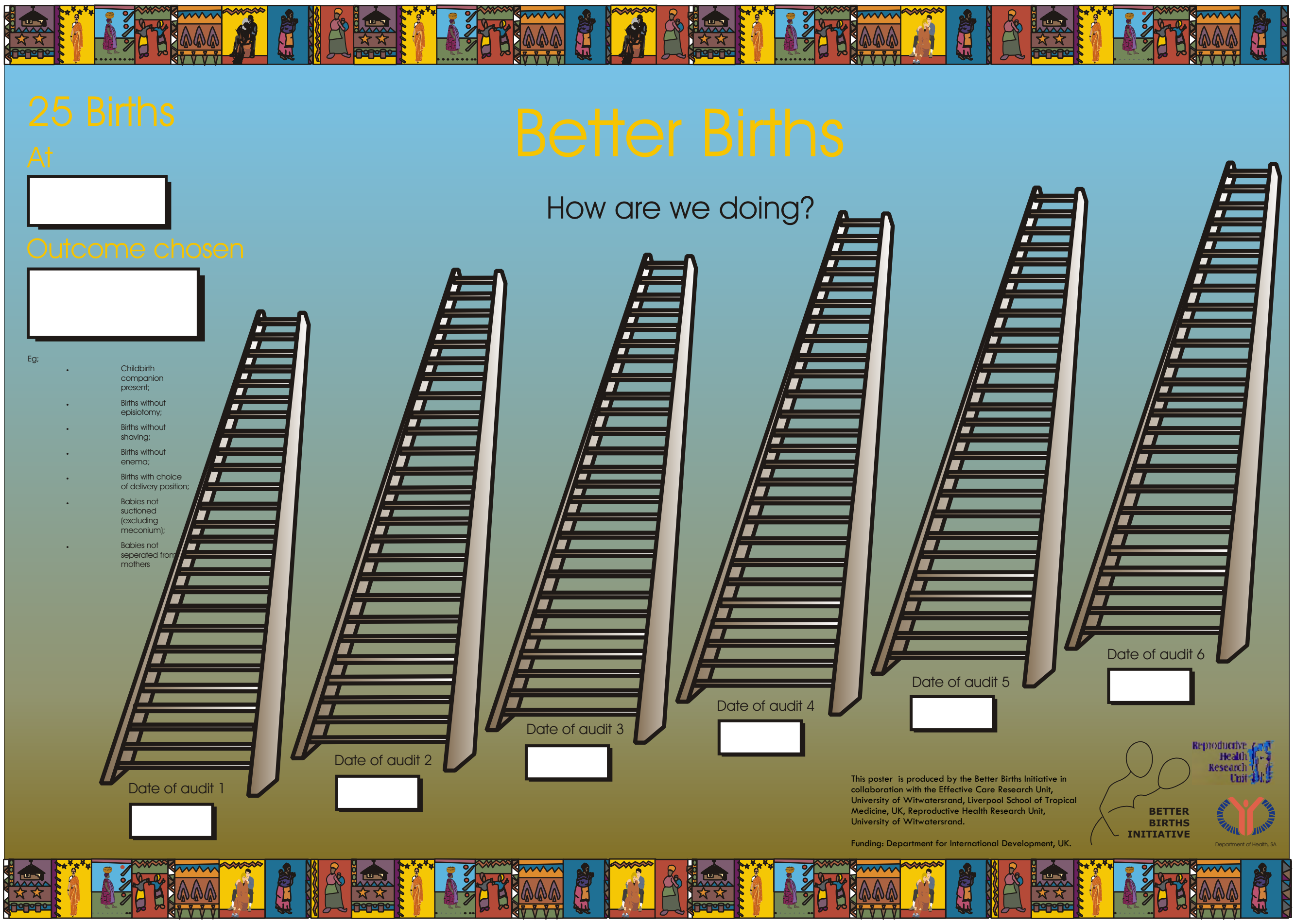
25 Births
At
Outcome chosen
Eg;
Childbirth companion present;
Births without episiotomy;
Births without shaving;
Births without enema;
Births with choice of delivery position;
Babies not suctioned (excluding meconium);
Babies not seperated from mothers
Better Births
How are we doing?
Date of audit 1
Date of audit 2
Date of audit 3
Date of audit 4
Date of audit 5
Date of audit 6
This poster is produced by the Better Births Initiative in collaboration with the Effective Care Research Unit, University of Witwatersrand, Liverpool School of Tropical Medicine, UK, Reproductive Health Research Unit, University of Witwatersrand.
Funding: Department for International Development, UK.
BETTER BIRTHS INITIATIVE
Reproductive Health Research Unit
Department of Health, SA
