# Supplementary material for: Promoting childbirth companions in South Africa: a randomised pilot study
Source: BMC Med. 2007 Apr 30;5:7. doi: 10.1186/1741-7015-5-7 (PMC1905915; doi:10.1186/1741-7015-5-7)
Supplement: Additional file 2 — A reference booklet for midwives detailing evidence associated with commonly used obstetric procedures; developed as part of the Better Births Initiative and published in 2001. Please note the systematic reviews underpinning the clinical procedures outlined in the materials could be out of date. We would recommend that the current evidence for each intervention or policy is reviewed before promoting practice based on these materials. For up to date Cochrane systematic reviews in sexual and reproductive health relevant to developing countries please refer to the current editions of either: the WHO Reproductive Health Library [6] or The Cochrane Database of Systematic Reviews [20]. [file 1741-7015-5-7-S2.pdf]

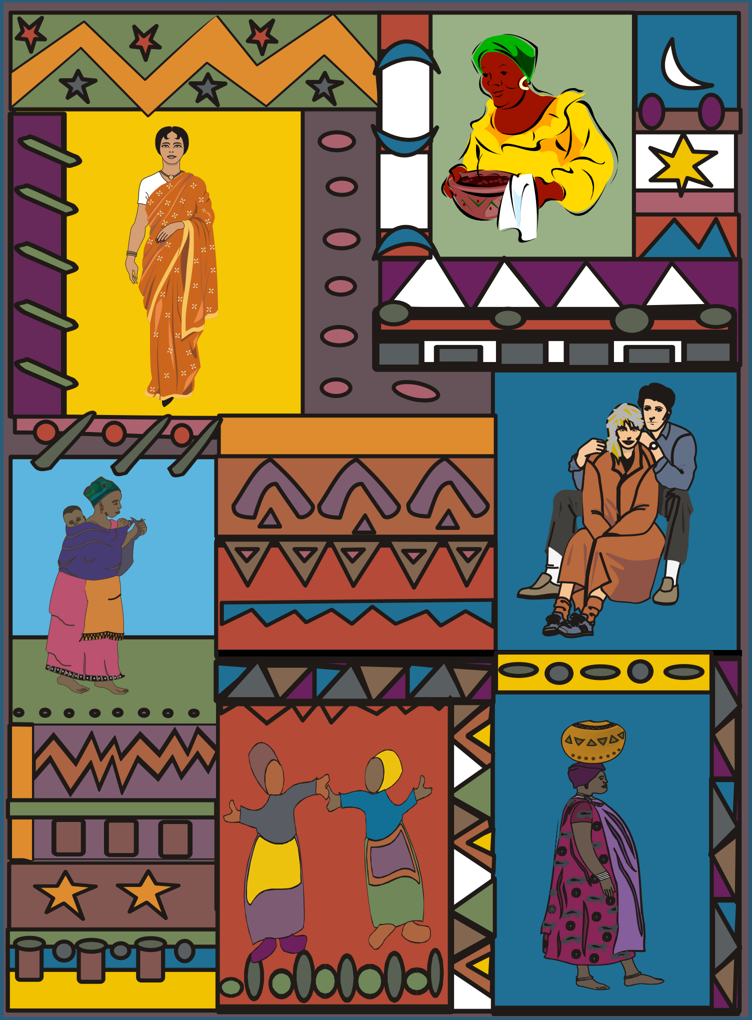

BETTER
BIRTHS
INITIATIVE

Making Childbirth Better

Reference Booklet

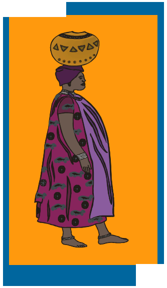

What is evidence-based maternity care?

As a provider of maternity care in a hospital or health centre, you will probably use a variety of procedures, checks and interventions to manage the labour process. Preparation for birth includes procedures like taking a women's blood pressure and checking her pulse and temperature. These are done routinely to ensure the health of the mother and enable a safe birth. However, there is evidence that some of the procedures used during labour do not actually offer any benefit to women.

Certain procedures are not always appropriate and can be harmful, unpleasant or uncomfortable for women.

Good care is based on procedures and interventions that have been justified by scientific research - in other words, evidence based care. As health professionals we must be aware of the most up to date research and change our practice accordingly.

Ideally, only interventions that are of benefit to women should be used, and those that are potentially harmful or humiliating should be stopped.

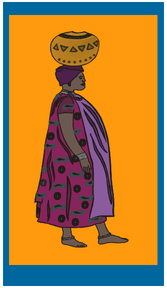

What information can I find in this reference booklet?

This reference booklet lists some commonly used procedures and the benefits and harms associated with them.

Keep this booklet handy; use it as a reference and always consider the potential benefits and harms of the procedures you use.

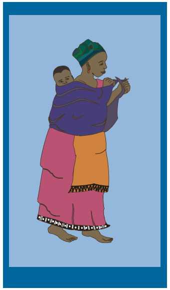

What is the evidence?

Mobility during labour

During the first stage of labour, you may prefer to restrict women to bed and have them lie in the supine position so that it is easier to conduct examinations and monitor progress. Several studies show that the supine position affects blood flow in the uterus, and can reduce the intensity of contractions. Standing or lying on the side can be more beneficial to the woman . As long as there are no complications, women should be encouraged to walk around, choose other more comfortable positions, and even take baths or showers during labour. No position is comfortable for a long period of time, encourage women to change to positions they feel most comfortable in.

There is no evidence to support the encouragement of strict bed rest during the first stage of labour. The only exception is when the membranes have ruptured in the presence of a non-engaged fetal head

Benefits	Harmful effects
• Standing or lying on the side are associated with greater intensity of contractions and labour progress.<br>• Labour maybe less painful if the woman is able to move around.<br>• Augmentation is less likely.	• There appear to be no harms associated with allowing women to move about freely and adopt different positions during the first stage of labour.

References:
1. Department of Reproductive Health and Research, WHO (1999). Care in Normal Birth: a practical guide. Geneva: World Health Organisation.
2. World Health Organisation. The WHO Reproductive Health Library, Issue 3, 2000. WHO/RHR/HRP/RHL/3/00. Oxford: Update Software

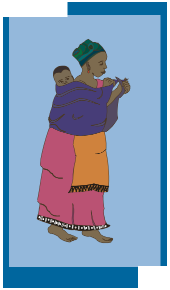

Different positions for delivery

During the second stage of labour, women are often confined to the supine or dorsal position to deliver. A number of studies suggest that an upright, or semi-upright position show more benefits than a supine position. Being upright is associated with less pain and discomfort, less vaginal trauma, and improved fetal outcome. Women often prefer a more upright position because they experience less pain and less backache compared to the supine position. Encourage women to adopt the position they find most comfortable, and avoid long periods lying supine.

Labour ward staff will need to become familiar with other positions for delivery, and be willing to attend women who prefer to adopt an upright or 'squatting' position. As a first step, the upright posture on the bed can be used while pushing, and the mother asked to lie back when the head 'crowns'

Benefits	Harmful effects
• Less discomfort in bearing down.<br>• Less labour pain.<br>• Perineal/vaginal trauma may be reduced.<br>• Possible reduction in labour duration.<br>• Improved Agpar score for baby	• Delivering in the upright position may increase the risk of blood loss.<br>• Possibility of increased risk of labial tears.

References:
1. Gupta JK, Nikodem VC. Woman's position during second stage of labour (Cochrane Review). In: The Cochrane Library. Issue 2, 2001.Oxford: Update Software
2. Department of Reproductive Health and Research, WHO (1999). Care in Normal Birth; a practical guide. Geneva: World Health Organisation.
3. World Health Organisation. The WHO Reproductive Health Library, Issue 4, 2001. WHO/RHR/HRP/RHL/3/00. Oxford: Update Software.

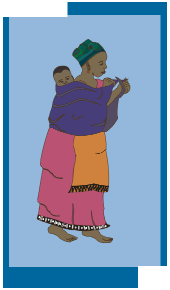

Fluids and food during labour

In many hospitals women are discouraged from eating and drinking during labour. The main reason for withholding food and fluids is fear of a spiration of gastric contents during general anaesthesia. This is a serious consideration. Restricting oral fluid and food intake, however, does not guarantee an empty stomach; and trials of various methods have shown that a 100% reduction of stomach content is not possible. Starvation in la bour does not protect women from aspiration of stomac h contents during general anaesthesia. This can only be achieved by using anaesthetic techniques to protect the airway (and using spinal anaesthesia whenever possible). Labour requires lots of energy and can last for hours - women need to maintain their energy levels. Restriction of oral intake can lead to dehydration and ketosis-this is usually treated with IV infusion. Some women may not want to eat during labour, but most will need to drink. Women are able to monitor their own intake, and will intuitively avoid heavy meals. For normal and low risk births, avoid interfering with women's wish for food and drink during labour.

Benefits	Harmful effects
- Energy levels are maintained.<br>- Dehydration is avoided.<br>- Complications due to a lack of food (ketosis) are avoided.	- Women may feel nauseous if they eat heavy meals during labour.

References:
1. Department of Reproductive Health and Research, WHO (1999). Care in Normal Bir th; a practical guide. Geneva: World Health Organisation.
2. Mckay S, Mahan C. Modifying the stomach contents of labouring women: why, how, with what success, and at what risks? How can aspiration of vomitus in obstetrics be prevented? Birth 1988;15 (4):213-221

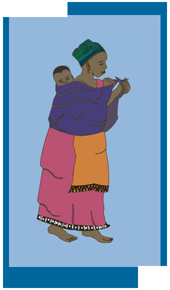

Encourage companionship during labour

There is evidence to suggest that women who are supported throughout labour by a partner, friend, relative or carer enjoy several benefits. Companions can provide emotional support, comfort, encouragement, physical contact, and a friendly face. There do not appear to be any harmful effects associated with support during labour. Encourage women to bring with them someone they trust or feel comfortable with: a partner, relative, or friend. Alternatively, establish a childbirth companionship system at your hospital/clinic.

Ideally, women need a private room in which to labour and deliver; somewhere that a male companion can be with them too. This may not always be possible in busy labour wards, but female supporters can usually be accommodated with minimal disruption.

Benefits	Harmful effects
• Labour may be shorter.<br>• Fewer interventions are needed during labour.<br>• Pain relief may not be necessary (drugs)<br>• Caesarean or assisted delivery is less likely<br>• Agpar scores may be higher<br>• Women who have support throughout labour often feel better about themselves, their labour and their baby.	• There do not appear to be any harms associated with continuous companionship during childbirth.

References:
1. Hodnett ED. Caregiver support for women during childbirth (Cochrane Review). In: The Cochrane Library. Issue 2, 2001.Oxford: Update Software
2. Department of Reproductive Health and Research, WHO (1999). Care in Normal Birth; a practical guide. Geneva: World Health Organisation.
3. Hofmeyr GJ, Nikodem VC, Wolman WL, Chalmers BE, Kramer T. Companionship to modify the clinical birth environment: effects on progress and perceptions of labour, and breastfeeding. British Journal of Obstetrics and Gynaecology 1991;98:756-764.
4. World Health Organisation. The WHO Reproductive Health Library, Issue 4, 2001. WHO/RHR/HRP/RHL/3/00. Oxford: Update Software.

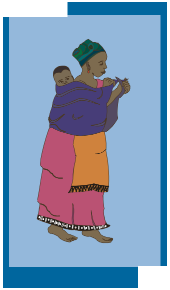

Magnesium sulphate for treating eclampsia

Eclampsia is a serious complication of pregnancy, causing an estimated 10% of maternal deaths per year world-wide. Eclampsia is treated with an anticonvulsant to control the initial fit and prevent further seizures. Diazepam (Valium), phenytoin, and lytic cocktail have been used to treat eclampsia. Magnesium sulphate, however, is the most effective treatment. Studies have shown it to be much more effective than other anticonvulsants, in addition it is relatively cheap and easy to use.

Magnesium sulphate should be used routinely to treat all women with eclampsia.

Benefits

Compared to other drugs, magnesium sulphate:

- is associated with a large reduction in the recurrence of convulsions.
- may reduce risk of maternal mortality
- may reduce likelihood of admission to intensive care.
- is cheap and easy to use.

Harmful effects

Careful monitoring is required because of the risk of respiratory depression

References:

1. Duley L, Henderson-Smart D. Magnesium sulphate versus diazepam for eclampsia (Cochrane Review). In: The Cochrane Library. Issue 2, 2001.Oxford: Update Software.
2. Duley L, Gulmezoglu AM. Magnesium sulphate versus lytic coctail for eclampsia (Cochrane Review). In: The Cochrane Library. Issue 2, 2001.Oxford: Update Software.
3.Duley L, Henderson-Smart D. Magnesium sulphate versus phenytoin for eclampsia (Cochrane Review). In: The Cochrane Library. Issue 2, 2001.Oxford: Update Software.

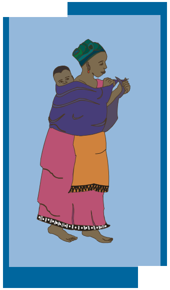

Oxytocin in the third stage of labour

Postpartum haemorrhage is significant cause of maternal deaths. Evidence shows that bleeding after birth can be reduced by the active management of the third stage of labour. This includes administering an oxytocic drug. Syntocinon 10 units intravenously or intramuscularly is almost as effective as syntometrine, and has the advantage of fewer side-effects, and being safe in women with hypertension.

Benefits	Harmful effects
Reduces blood loss after birth	Requires intramuscular or intravenous injection

References:

1. World Health Organisation. The WHO Reproductive Health Library, Issue 4, 2001. WHO/RHR/HRP/RHL/3/00. Oxford: Update Software.

Strategies to reduce mother to child transmission of HIV

Increasing numbers of children are becoming infected with the human immunodeficiency virus (HIV) by their mothers. The risk of mother-to-child transmission (MTCT) is estimated to be 25-35% in Africa. There are several interventions that can effectively reduce the risk of MTCT- some are discussed here, with particular emphasis on those strategies that are applicable to low-income settings.

Breast feeding

If women are in a position to provide safe alternative feeding, they may be encouraged not to breastfeed. If they need to or wish to breastfeed, they should be encouraged to give their baby ONLY breast milk for about 4 months, then change to a mixed diet and stop breastfeeding.

Elective caesarean section

Evidence suggests that caesarean delivery can reduce mother-to-child transmission of HIV. However, in low-income settings caesarean delivery can put the mother at risk, and it is recommended that, if available, short course Nevirapine or zidovudine, be used instead.

Vitamin and nutritional supplements

Studies in developing countries have suggested that the risk of mother-to-child transmission of HIV infection is associated with vitamin A deficiency in the mother. It is possible that vitamin A supplementation will decrease this risk, but further studies are required to determine the effectiveness of this strategy.

Vaginal cleansing

Most mother-to-child transmission is thought to occur at the time of delivery, and it is suggested that disinfecting the vagina before and/or during labour may help to prevent transmission. Disinfective agents, such as Chlorhexidine, may be particularly useful because of their activity against HIV. More clinical trials need to be conducted to determine the effectiveness of this procedure.

Antiretrovirals

Combination therapy (use of three or more drugs together) can delay the emergence of drug resistance and is now widely used in high-income countries. In low-income settings effective strategies, that are cheaper and simpler, are available:
Nevirapine is an antiretroviral drug that is rapidly absorbed when given orally and has very strong activity against the virus. It is possible to provide this effective antiretroviral during labour and delivery with a single oral dose. Nevira pine one tablet to the mother during labour, and one dose of syrup to the baby after birth, is highly effective in reducing the risk of transmission. Nevirapine is much cheaper than zidovudine. Zidovudine is another antiretroviral drug that prevents the virus reproducing, and is effective in reducing the risk of transmission. There do not appear to be any adverse effects in infants up to the age of 4 years.

Artificial rupture of membranes

Rupture of membranes has been identified as a potential risk factor for mother-to-child transmission of HIV infection. Avoiding artificial rupture of the membranes (ARM) may be an effective and simple way of reducing the risk of transmission. Avoiding ARM may, however, result in other difficulties such as prolonged labour, which may increase the risk of transmission occurring. Further clinical trials are required to assess the benefit of avoiding or delaying ARM.

References:
1. Brocklehurst P. Interventions aimed at decreasing the risk of mother-to-child transmission of HIV infection (Cochrane Review). In: The Cochrane Library, Issue2, 2001. Oxford: Update Software.
2. Dabis F, Msellati P, Newell ML, Halsey N, Van de Perre P, Peckham C et al. Methodology of intervention trials to reduce mother-to-child transmission of HIV with special reference to developing countries. AIDS 1995;9 Suppi A:S67-S74.
3. Dunn D, Newell M-L, Ades A, Peckham C. Risk of human immunodeficiency virus type 1 transmission through breast-feeding. Lancet 1992;240:585-8.
4. Landesman SH, Kalish LA, Burns DN, Minkoff H, Fox HE, Zorilla C et al. Obstetrical factors and the transmission of human immunodeficiency virus type 1 from mother to child. New England Journal of Medicine 1996;334:1617-23.
5. Semba RD, Miotti PG, Chiphangwi JD, Saah AJ, Canner JK, Dallabetta GA et al. Maternal vitamin A deficiency and mother-to-child transmission of HIV-1. Lancet 1994;343:1593-7.
6. World Health Organization. The WHO Reproductive Health Library, Issue 4, 2001. WHO/RHR/HRP/RHL/3/00. Oxford: Update Software.

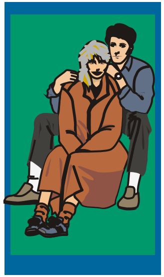

Reduce use of early amniotomy

Artificial rupture of the membranes has been widely practised for several decades as a component of the active management of labour. It was thought that ARM might reduce the risk of caesarean section. More recently, studies have looked at delaying ARM, or conserving the membranes. Routine early amniotomy is associated with both benefits and harms. Benefits include reduced length of labour, and a possible reduction in abnormal 5-minute Agpar scores. Until there is more evidence, amniotomy should be reserved for women with abnormal or slow progress.

In addition, ARM is a potential risk factor for mother-to-child HIV transmission (see page 10)

Benefits	Harmful effects
• Reduced length of labour.<br>• Possible reduction in abnormal Agpar scores	• Potential risk of mother-to-child HIV transmission.

References:
1. Fraser WD, Turcot L, Krauss I, Brisson-Carrol G. Amniotomy for shortening spontaneous labour (Cochrane Review). In: The Cochrane Library. Issue 2, 2001.Oxford: Update Software.

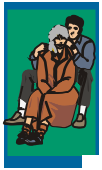

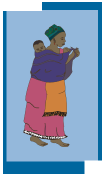

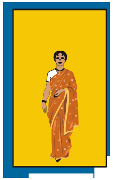

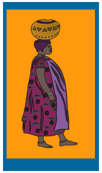

Reserve suctioning for babies with meconium present

Routine nasopharyngeal suctioning of all newborns is unnecessary, and costly. There is a risk of aspiration complications when suctioning is performed unnecessarily. It is also thought that suctioning may cause abrasions that increase the risk of transmission of HIV to the baby. It is recommended that only those babies with meconium present should undergo suctioning on delivery.

Benefits	Harmful effects
• There are benefits associated with suctioning babies WITH meconium present	• Risk of aspiration complications in newborns without meconium present.<br>• Risk of HIV infection

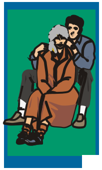

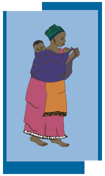

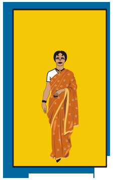

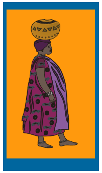

Enemas are not always necessary

Enemas are still widely used on admission to prepare women for childbirth. The use of enemas may be unnecessary in all cases. Enemas can be uncomfortable for women, and the procedure is an additional expense. Without an enema, faecal soiling is more solid and easier to remove than soiling after an enema. There is insufficient evidence to recommend the routine use of enemas and further medical trials need to be conducted before the benefits and harms can be properly evaluated. An enema should be given only if the women requests it.

Benefits	Harmful effects
It is believed that:<br>• an empty bowel helps the head to descend.<br>• enemas may reduce contamination and therefore reduce the chance of infection<br>There is no evidence to suggest that this is true.	• Enemas may be uncomfortable and embarrassing.<br>• There is risk of damage to the bowel.

References:
1. Cuervo LG, Rodriguez MN, Delgado MB. Enemas during labour (Cochrane Review). In: The Cochrane Library. Issue 2, 2001.Oxford: Update Software.
2. Department of Reproductive Health and Research, WHO (1999). Care in Normal Birth; a practical guide. Geneva: World Health Organisation.
3. World Health Organisation. The WHO Reproductive Health Library, Issue 4, 2001. WHO/RHR/HRP/RHL/3/00. Oxford: Update Software.

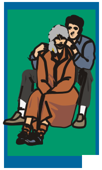

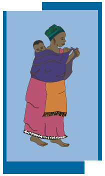

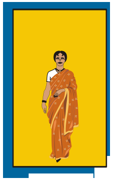

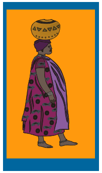

Stop pubic shaving

In the past, pubic shaving in preparation for birth was a routine procedure and it was believed to have many benefits. Now it is widely recognised that shaving women is an unnecessary procedure. If you stop shaving, you will reduce the discomfort and embarrassment for women, and reduce the risk of HIV infection for both women and staff.

Benefits

It is believed that shaving can:

- reduce infection with tearing or episiotomy
- make the stitching of tears or cuts easier.

But there is no evidence to suggest that this is true.

Harmful effects

- Some women feel embarrassed to have their pubic hair shaved.
- Some women experience a lot of discomfort when the hair grows back.
- Other side effects include: redness, multiple scratches, and burning and itching of the vulva.
- If shaving is done routinely, with a non-sterile blade, the risk of HIV infection may be increased

References:
1. Basevi V, Lavender T. Routine perineal shaving on admission in labour (Cochrane Review). In: The Cochrane Library. Issue 2, 2001.Oxford: Update Software.
2. Department of Reproductive Health and Research, WHO (1999). Care in Normal Birth; a practical guide. Geneva: World Health Organisation.
3. World Health Organisation. The WHO Reproductive Health Library, Issue 4, 2001. WHO/RHR/HRP/RHL/3/00. Oxford: Update Software.

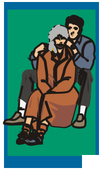
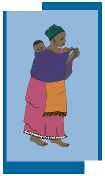
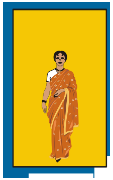
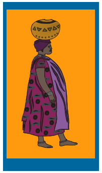

Avoid episiotomy

Cutting the skin and muscle that surrounds the va gina to allow the baby to be born is a procedure that is used routinely in some parts of the world, par ticularly for first bir ths. Midwives and doctors justify the use of episiotomy because it is thought to prevent damage to the anal sphincter and rectum, is easier to suture and allows be tter healing than a natural tear. Episiotomy carries a number of risks, and the evidence suggests that a routine use does not reduce perineal trauma or improve healing. There is clear evidence to recommend r estricted use of episiotomy. In addition, staff performing episiotomy are at risk of HIV infection.

Benefits

There is no evidence of any benefits associated with routine use of episotomy, but it may be used in the following circumstances:

- to prevent serious third degree tearing
- if the ba by is in distress
- if the la bour is not progressing

Harmful effects

- Perineal trauma.
- Healing complications (infection).
- Pain for a long period after birth.
- Dyspareunia (painful intercourse)
- Delayed breast feeding if the women cannot sit upright while the cut is healing.
- Risk of ver tical transmission of HIV.

References:
1. Carroli G, Belizan J. Episiotomy for vaginal birth (Cochrane Review). In: The Coc hrane Library. Issue 2, 2001.Oxford: Update Software.
2. Department of Reproductive Health and Research, WHO (1999). Care in Normal Bir th; a practical guide. Geneva: World Health Organisation.
3. World Health Organisation. The WHO Reproductive Health Library, Issue 4, 2001. WHO/RHR/HRP/RHL/3/00. Oxford: Update Software.

What is the 'Better Births Initiative'?

BETTER
BIRTHS
INITIATIVE

It is important for health care providers to continually improve the quality of care, make it more humane, and make the experience of labour more comfortable. This is in itself a worthwhile goal. It will also enhance the reputation of the service and encourage women to attend.

Greater use of services is a key step in reducing the half a million maternal deaths in developing countries each year.

The Better Births Initiative is a focused set of standards that aim to improve the quality and humanity of obstetric care. The standards are based on the best available evidence, and can be implemented using existing resources.

We hope you will work towards 'Better Births' in your labour ward.

BETTER BIRTHS INITIATIVE

Principles of the Better Births Initiative:

Humanity	women treated with respect.
Benefit	care that is based on the best available evidence.
Commitment	health professionals committed to improving care.
Action	effective strategies to change current practice.

REPRODUCTIVE HEALTH RESEARCH UNIT

RHRU

RHRU is a research unit of the University of the Witwatersrand, South Africa.

Department of Health

Private Bag X828, Pretoria, 0001

Acknowledgements

'This booklet is produced by the Better Births Initiative, a Collaboration between: the Effective Care Research Unit, University of Witwatersrand; Liverpool School of Tropical Medicine; Reproductive Health Research Unit, University of Witwatersrand.'

Funding: Department for International Development, UK
SA Medical Research Council
International Childbirth Education Association

Authors

Helen Smith, Heather Brown, Justus Hofmeyr,
Kim Dickson Tetteh, Paul Garner, Helen Rees

© Copyright protected

Effective Care Research Unit

BETTER BIRTHS INITIATIVE

BETTER BIRTHS INITIATIVE

Contact details

Better Births Initiative
℅ Reproductive Health Research Unit
Department of Obstetrics and Gynaecology
Chris Hani Baragwanath Hospital
PO Box Bertsham 2013
Johannesburg
South Africa
Phone: 011 933 1228
Fax: 011 933 1227
Http://www.liv.ac.uk/lstm/bbimainpage.html

Layout and Design: RHRU Marketing & IEC

Published: March 2001

BETTER BIRTHS INITIATIVE
